# Acoustic Signatures in Laser-Induced Plasmas for Detection of Explosives in Traces

**DOI:** 10.3390/s26020672

**Published:** 2026-01-20

**Authors:** Violeta Lazic, Biljana Stankov, Fabrizio Andreoli, Marco Pistilli, Ivano Menicucci, Christian Ulrich, Frank Schnürer, Roberto Chirico, Pasqualino Gaudio

**Affiliations:** 1 Italian National Agency for New Technologies, Energy and Sustainable Economic Development (ENEA), Laboratory NUC-TECFIS-DIM, Via Enrico Fermi 45, 00044 Frascati, Italy; 2 Institute of Physics Belgrade, University of Belgrade, 11080 Belgrade, Serbia; biljanas@ipb.ac.rs; 3 Italian National Agency for New Technologies, Energy and Sustainable Economic Development (ENEA), Laboratory NUC-FUSEN-TEN, Via Enrico Fermi 45, 00044 Frascati, Italy; 4 Fraunhofer Institute for Chemical Technology ICT, Energetic Materials Department, Joseph-von-Fraunhofer-Str. 7, 76327 Pfinztal, Germany; 5 Department of Industrial Engineering, University of Rome Tor Vergata, Via del Politecnico 1, 00133 Roma, Italy

**Keywords:** explosives, traces, laser, acoustic, LIBS, detection, forensic, sensitive, limit of detection

## Abstract

In this work we report the results of analysis of the acoustic signal generated by the interaction of a nanosecond laser pulse (30 mJ, 1064 nm) with various residues placed on a silica wafer. The signal was captured by a unidirectional microphone placed 30 mm from the laser-generated plasma. The examined sample classes, other than the clean wafer, included particles from soils and rocks, carbonates, nitro precursors, ash, coal, smeared diesel, and particles of explosives. We tested three types of explosives, namely PETN, RDX, and HMX, having different origins. For the explosives, the acoustic signal showed a faster rise, larger amplitude, different width, and attenuation compared with the other sample classes. By subtracting the acoustic signal from the wafer at the same position, obtained after four cleaning laser pulses, the contribution of echoes was eliminated and true differences between the residue and substrate became evident. Through four different features in the subtracted signal, it was possible to classify explosives without the presence of false positives; the estimated limit of detection was 15 ng, 9.6 ng, and 18 ng for PETN, RDX, and HMX, respectively, where the mass was extrapolated from nano-printed samples and LIBS spectra acquired simultaneously. Furthermore, HMX was distinguished from the other two explosives in 90% of the cases; diesel and coal were also recognized. We also found that explosives deposited through wet transfer behaved as inert substances for the tested masses up to 30 ng.

## 1. Introduction

Explosives are characterized by their potential to rapidly release large amounts of energy in the form of high temperatures, intense pressures, and supersonic shock waves. These materials are broadly classified based on their physical and chemical characteristics; common examples include high-energy explosives, such as hexogen (RDX), octogen (HMX), trinitrotoluene (TNT), and pentaerythritol tetranitrate (PETN), and low-energy explosives like black powder or flash powder [1]. Explosives can also be categorized according to their chemical composition into pure substances and mixtures. These substances may exist in various chemical forms, including particulates, residues, liquids, emulsions, vapors, or aerosols. Due to their destructive potential, the detection of explosives remains a focal point in security, forensic, and environmental safety. Most of the standard solid explosives exhibit very low vapor pressure at room temperature [2], thus their trace detection is limited to the particle form. Presently, the collection of trace amounts of explosives is mostly carried out by swab materials (glass fiber, cotton, polymers, etc.) or by pre-concentrators based on forced air flow.

Mass spectrometry (MS) offers high levels of sensitivity and specificity compared with other technologies for chemical detection, and for pure substances the detection limits by MS are below ng levels [3]. MS is often coupled with upstream separation techniques, such as liquid chromatography (LC) or gas chromatography (GC), used to isolate compounds before analyzing them in detail [4,5]. The traditional disadvantages of MS instruments are high cost and complexity, including the use of a vacuum system. Recent developments have brought direct analysis in real time DART-MS, which has become capable for routine and direct analysis of samples at atmospheric pressure [6]. This technique is viable for the screening of trace explosives on different substrates; for the measurement time of 10 s, the reported detection limits are between 2.5 and 50 ng, depending on the sample and substrate [7].

Ion mobility spectrometry (IMS) is similar in concept to MS except that the ions are dispersed by gas-phase viscosity and not by molecular weight; the method does not require vacuum operation. Nowadays, compact, low consumption, and easy to use IMS devices are widely employed in airport security checks, although they represent the trade-off because of low resolving power and limited selectivity for explosive residues [8]. IMS sensitivity is generally in the range 1–100 ppb or 0.01–10 ng for IMS sensitive compounds [9].

Raman spectroscopy is a laser-based non-destructive technique capable of directly detecting and identifying pure substances, without sample preparation, in a large variety of chemical materials, including explosives [10]. By using proper equipment, the detection of explosives is viable also at a distance [11,12]. When probing trace materials, the sensitivity also depends on the substrate; in the case of a non-fluorescing substrate, RDX traces of about 150 ng mass are detected at a distance of 10 m [12]. The instruments for stand-off detection are not commercially available, compared with handheld Raman spectrometers that have become widely used. However, due to an inherently low Raman signal, these small instruments usually require bulk samples or their mass in mg range, which, in the case of some substances, poses a hazard of laser-induced ignition [13]. Surface-enhanced Raman spectroscopy (SERS) allows to analyze trace materials, including explosives, thanks to the increased sensitivity through special nanostructured metal-containing substrates [14]. By using SERS, a successful detection of TNT collected from fingerprints by swabbing, was recently reported [15]. The drawback, besides the limits inherent to Raman techniques, is related to the costs and lifetime of the fabricated SERS substrates. To overcome this limitation, much research effort is placed into the development of flexible SERS substrates, starting with papers, fabrics, polymer nanofibers, and cellulose [16].

Other devices for the detection of explosives in traces are based on artificial olfaction [17], which, however, still need further developments to overcome existing challenges and gain acceptance among the other commonly used techniques. While mass spectrometry remains the reference and the most accurate method for the detection of explosives, there is a necessity to improve the existing devices or develop new ones that could match the requirements for rapid screening and wide use, e.g., short analysis time, very low false-positive rate, high sensitivity, easy to use and interpret by non-specialists, and, finally, low purchase and operating costs. For these reasons, the existing techniques and instruments for the in situ rapid detection of explosives in traces are periodically reviewed and critically discussed to point out the limits and gaps that need yet to be solved [18,19,20,21,22,23].

One of the emerging techniques in security and forensic examinations is laser-induced breakdown spectroscopy (LIBS), which provides information about elemental sample composition; it also has stand-off detection capabilities allowing the examination of hazardous materials from a safe distance. LIBS instruments with high-resolution detectors covering a large spectral range can provide multi-elemental analysis by a single laser pulse, with the detection sensitivity below 10 pg per element [24]. The latter feature is of particular interest in the analysis of trace sample materials, as in the case of forensic examinations and rapid screening. For measurements surrounding air, where nitrogen, oxygen, and hydrogen, are present, the recognition of explosives by LIBS can be achieved by various discrimination strategies, e.g., comparing the intensity ratios of atomic or molecular transitions or applying chemometric data analysis methods [25,26,27,28,29,30,31]. Although the identification of explosives is difficult to achieve by LIBS, their rapid detection at sub-ng levels at close distances [28,30] or in ng quantities at a distance of 10 m [12] with low rates of false positives makes this technique extremely interesting. Furthermore, the integration of Raman and laser-induced fluorescence (LIF) techniques into a single instrument [12] increases the possibilities of high-sensitivity elemental and molecular characterization of materials.

When a high-energy laser pulse, typical for LIBS, interacts with a solid target, it induces plasma formation accompanied by a shockwave. This wave expands into the surrounding medium, slowing down with distance until reaching the speed of sound of the medium. In fixed experimental conditions, the plasma-generated acoustic signal is sensitive to material hardness, chemical composition, and optical properties, thus supplying additional information about the sample [32,33]. The acoustic signal also represents a good reference signal for spectral normalization, exploited to improve the LIBS detection reproducibility and accuracy [33,34].

The acoustic signal captured by the microphone at a distance of 1–15 cm is used to characterize a laser-generated shockwave on a copper plate, showing that this method could be used for a microscale energetic characterization, providing relations between laboratory and free-field detonation testing of explosives [35]. Following the ablation of a thin layer of energetic powder with a mass in order of 10–100 µg, it is found that exothermic reactions influence the laser-induced shock wave due to the initial plasma and subsequent combustion reactions of ejected particles [36]. Characterization of the shock wave propagation after the laser-induced air shock for energetic materials (LASEM), where the ablated mass per pulse is in order of 0.1–1 mg, represents a rapid, cost effective, and safe way to measure performances of explosives, such as detonation velocities and chemical energy [37]. Another approach utilizes acoustic emissions during the laser ablation of explosives, employing an MHz bandwidth vibrometer to probe near-field density changes in the resulting acoustic waves [38]. The authors found that explosives were generating high ultrasound frequencies and asymmetric pressure waves with significantly larger amplitudes compared with inert materials, allowing to discriminate explosives with a detection limit of about 100 ng∕cm^2^.

In the present work, the laser-induced plasma was generated on small amounts of sample materials placed on silica wafers. The material classes examined, besides clean substrate, included carbonates, soils and grounded rocks, precursors based on nitro compounds, ash, coal particles, diesel residues, and three types of explosives (PETN, HMX, and RDX) from different proveniences. We studied various features relative to the acoustic signal captured by a microphone to find those that distinguished explosives from the other sample classes. The detection limits for explosives were extrapolated by using nano-plotted samples and the line intensities from carbon and silica in the LIBS spectra simultaneously captured with the acoustic signal.

## 2. Materials and Methods

In the following, we describe the sample set involved in testing and calibration, and the experimental set-up used for signal generation and detection. Although both the acoustic and LIBS signals were always acquired simultaneously, the focus of this work is on the discrimination of explosives through the acoustic signal, although some features of the LIBS spectra were exploited only for estimating the detection limits.

### 2.1. Samples

The substrate material was silica wafers 0.55 mm thick, covered by a SiO_2_ layer of 285 nm. The original wafers had diameters of 4 inches, and were cut into smaller rectangular pieces by a diamond cutter. To avoid surface contamination, the operator used nitril gloves; the residual particles from the cutting were blown by clean dry air. The advantage of using a Si wafer substrate consists in its stability over time, high purity, and enhancement of the plasma emission from particles [39] or thin liquid films [40]. Clean substrate, considered as a Class 1 material, was probed during all measuring sessions and compared with wafers loaded with various residues.

Particles of different sample types were delivered to the substrates and repeated single-shot measurements in different positions were performed by varying the surface coverage inside the laser spot from full (analog to probing of bulk material) to a very few particles. This operation was facilitated by live images supplied by the color camera of the LIBS instrument and overlapped via software by a circle corresponding to the laser spot. Frequently, it was necessary to add new particles to the substrates due to their blowing by laser induced shockwaves.

The Class 2 particles here corresponded to three soil samples and one powdered rock material, all of them with certified characteristics. The sample Class 3 regards carbonate mixtures was prepared in the laboratory, namely CaCO_3_:BaCO_3_ and CaCO_3_:LiCO_3_ in mass proportions of 50:50 and 90:50, respectively. Among nitrates, attributed to Class 4, we tested laboratory grade KNO_3_ and urea nitrate, which were in the form of powder or fine crystals. To compare carbon rich materials of similar elemental composition but with very different burning potential, we considered coal fly ash (Class 5) and bituminous coal (Class 6). For coal, the certified calorific value was 31.9 ± 0.24 MJ/kg. Diesel residues (Class 7) were smeared on wafers and probed at points with various local film thicknesses. All above mentioned materials were compared with particles of explosives having two or three different origins, namely PETN (Class 8), RDX (Class 9), and HMX (Class 10).

The full sample set tested is summarized in Table 1, together with the set used to evaluate the mass of particles from explosives inside the laser spot.

For mass calibration, we used nano-plotted dots of PETN and RDX with the known mass per spot between 1 ng and 30 ng. For the highest mass load, the printed spot had a diameter of about 200 µm, the same size as the laser spot diameter. Explosives diluted in acetonitrile (PETN: 15 and 1.15 mg/mL, RDX: 5 and 1.15 mg/mL) were used for delivering spots on wafers by drop-on-demand printing, performed at Fraunhofer ICT with a Nanoplotter NP 2.1 from GeSIM. The printer was equipped with a piezo pipette of the “Nanotip” type, delivering droplet sizes of 280–320 pL, whose volumes were then measured by a stroboscopic view and flow sensor. Electric impulses (87 V, frequency 100 Hz) delivered single droplets from the orifice at the tip of the piezo pipette. An automatic X-Y-Z stage released one or multiple droplets of printing solution on defined locations of the flat substrates. After the evaporation of the solvent, solid deposits remained on the surface. For studies of the detection limits, the spots with PETN or RDX between 1 ng and 30 ng were prepared by applying 3–21 droplets on silica wafers, in a grid of 4 × 4 spots distanced 2 mm from each other. These spots were later precisely selected by the X-Y-drive of the LIBS instrument.

### 2.2. Laser and Detection System

The experimental set-up was based on the LIBS instrument initially developed for the detection of trace elements at a crime scene, and its full description and performances are reported in a previous work [24].

The laser source for generating the plasma on samples was a diode-pumped Nd:YAG laser by BrightAerospace (Pavia, Italy), model BAS_CDL-1064-50mJ-DB. The laser emitted pulses of 6.5 ns duration at a wavelength of 1064 nm, with a repetition rate between 1 Hz and 5 Hz. The measurements on residues were performed by five consecutive pulses, with an incident energy of 30 mJ and a repetition rate of 3 Hz. The laser was externally triggered, where time 0 corresponded to the beginning of the laser pumping, while the Q-Switch opening, i.e., emission of the laser pulse, was delayed for 200 µs from the first trigger. In the LIBS measurements, the spectrometers were opportunely pre-triggered to compensate for an inherent electronic delay of the detectors and guaranteed that the acquisition delay was effectively varied with respect to the laser pulse (delay 0).

The LIBS instrument sensor head was pre-aligned for placing the target surface at a distance of 12.6 mm from the exit, in a way to generate the laser spot of an equivalent diameter of 0.30 mm. In such conditions, for the incident laser energy of 30 mJ, the energy density on the target was about 42 J/cm^2^, corresponding to an instantaneous power density of 6.5 GW/cm^2^. The optically collected plasma emission was spectrally resolved and detected by six compact spectrometers by Avantes, Apeldoorn, The Netherlands (model AvaMINI 2048), covering a range from 180 nm to 875 nm. The detectors had a minimum integration time of 30 µs, here used for the LIBS signal acquisition. In the present experiment, we exploited the LIBS signal only from the first two spectrometer channels, with a spectral resolution of about 0.05 nm, to monitor C I and Si I lines and evaluate the detection limits on explosives obtained from the acoustic signal.

The acoustic signal was captured by a common unidirectional microphone (CUI Devices, Portland, OR, USA, model CMI-5247TF-K) with a diameter of 9.7 mm. The shielded microphone was encapsulated in a plastic box with a pinhole 1 mm in diameter and covered by a 5 mm thick sponge layer. The microphone had a built-in low-noise JFET preamplifier and was powered by 1.5 V to limit electrical noise. The microphone was placed below the LIBS instrument head, at a distance of 30 mm from the laser spot on the target and at an angle of about 18° with respect to the target plane. This distance was necessary to avoid the receiver’s saturation by intense pressure waves induced by the plasma–target interaction. Acquisition of the acoustic signal by an oscilloscope (Rohde&Schwarz, Munich, Germany, model RTA4K) was triggered together with the laser pumping (200 µs before the laser pulse), where the sampling rate was set to 10 MHz.

For visualization and positioning of residues on the target, we exploited the color camera built in the LIBS instrument, providing the theoretical spatial resolution of 1.5 µm. The instrument’s software superimposed the laser spot on the target, represented as a circle, on the live images provided by the camera. In this way, the selection of areas containing the sample residues was facilitated. Before the laser pulse sequence was launched via the instrument’s graphical user interface (GUI), the last frame before the laser shooting was automatically stored; additional photos, for example after the measurement, could also be taken. The sample could be illuminated by turning on an array of white LEDs placed inside the instrument head.

## 3. Results and Discussion

This section presents a comprehensive analysis of the acoustic responses generated by laser irradiation on trace amounts of explosives and combustible and inert materials deposited on a silica wafer. Through the images of the target acquired before and after the laser pulses, we monitored the initial amount of sample inside the laser spot and progressive surface cleaning by the laser pulses. The LIBS spectra simultaneously acquired with the acoustic signal were exploited here to estimate the mass of explosives involved in the plasma and acoustic signal formation.

### 3.1. Signal from Microphone

The laser pulse emittance at 200µs from the electrical trigger was accompanied by a short electrical disturbance of the microphone, even though it was connected to the oscilloscope with the electrically shielded cable. For the microphone distanced about 30 mm from the plasma induced on the wafer, the sharp rise of the acoustic signal due to the arrival of the pressure wave occurred after about 84 µs from the pulse, with energy of 30 mJ. The shape of the acoustic wave corresponded to what has already been observed in other laser ablation experiments [34,37,41], where the first and most prominent positive peak is followed by a slow recovering negative signal and occurrence of echoes from nearby surfaces. For laser ablation with irradiance in order of GW/cm^2^ higher, the initial shockwave has supersonic speed dependent on the laser irradiance and sample properties, and it rapidly attenuates with distance, while the width of the pressure peak enlarges [37,41]. The ablation of materials that pass rapid oxidation, like explosives, increases the plasma temperature and, consequently, shockwave speed and acoustic amplitude; on the other hand, self-sustained combustion produces a delayed acoustic signal [36,37].

**Figure 1:**
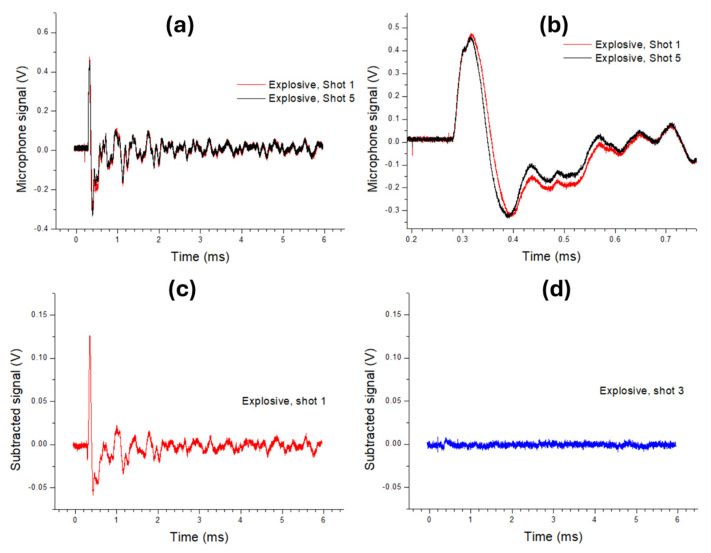
Comparative signal from microphone obtained from explosive particles from wafer (1st shot) and clean substrate (5th shot) at a large scale (**a**) and zoomed (**b**). Signal from explosive from the 1st shot (**c**) and 3rd shot (**d**) after subtracting the contribution from clean wafer (5th shot); the data were smoothed over 20 points. Laser energy was 30 mJ.

Comparative signals obtained from particles from an explosive probed by the first laser pulse, and the fifth pulse at the same location that had already hit the cleaned wafer surface, are shown in Figure 1a,b. On a large time scale, the acoustic signal showed an initial peak and valley, followed by a progressively weaker oscillation for another few milliseconds, where the features were also affected by the acoustic reflection from nearby surfaces and objects (instrument head and inner components, microphone holder, sample and its support). By observing the details (Figure 1b), in the presence of the explosive particles, the first acoustic peak had a larger amplitude and duration compared with the clean wafer. The negative peak was close to 0.4 ms, and, in the presence of an explosive generated under pressure, was larger up to about 0.65 ms.

The contribution of explosive particles to the acoustic signal was clearly visible after the subtraction of the signal from the clean wafer, considered to correspond to the fifth laser pulse (Figure 1c). The largest differences were observed during the first oscillation cycle, so further studies were focused on the timelapse up to 0.75 ms from the electrical trigger, i.e., to 0.55 ms from the laser pulse. In Figure 1d we also show the subtracted signal after the third laser shot at the same position—this signal was flat, indicating that the previous two laser pulses completely cleaned the wafer surface.

For the same microphone distance, observable also through the arrival time of the pressure wave at a fixed laser energy, the signal amplitude was very sensitive to changes in the microphone’s orientation [41]. Furthermore, due to acoustic reflections from nearby mechanical components, the signal shape also depended on the position of the sample holder and on the centering of the wafer piece on the holder.

**Figure 2:**
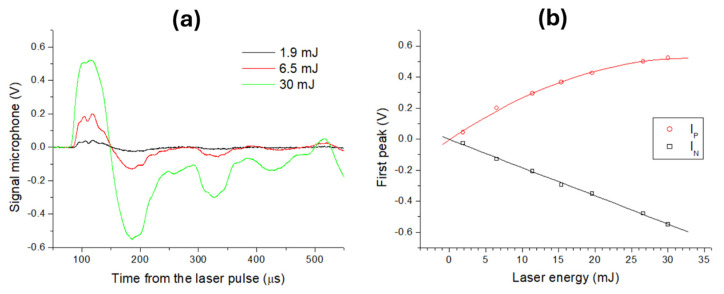
(**a**) Signal behavior on wafer for laser pulses with different energies; the data were smoothed over 20 points. (**b**) Values of the first maximum (red, polynomial fit) and the first minimum (black, linear fit) as a function of the laser pulse energy.

After the microphone re-orientation to maximize the acoustic amplitude, we studied signal behavior on the clean wafer by changing the laser energy, and some examples are shown in Figure 2a. We calculated the wave arrival time *t_a_* (µs) to the microphone as corresponding to 10% of the first maximum I_P_ (V), where the signal was first leveled to zero bias and smoothed by adjacent averaging over 20 points. With an increase in the laser energy from 1.9 mJ to 30 mJ, the *t_a_* was progressively reduced from 87.7 µs to 83.6 µs. For the pulse energy of 1.9 mJ, no visible plasma was formed, so the weak detected sound could be attributed to rapid laser-induced heating of the substrate/air. Considering that the speed of sound in air is 344 m/s, the effective distance between the microphone and the plasma center was 30.2 mm. Based on this value, the shockwave speed averaged over a distance of 30.2 mm was 360.7 m/s for the clean wafer irradiated by 30 mJ laser pulses. The width of the first pressure wave, calculated between *t_a_* and the successive signal decay to zero, was constant inside the measuring error, with a value of 63.5 ± 0.7 µs for the laser pulse energies between 1.9 mJ and 30 mJ.

On the wafer, the first positive peak I_p_ showed saturation, with an increase in the laser pulse energy, while the negative peak I_N_ had a linear behavior (Figure 2b). From this point of view, searching for differences in laser–sample interactions through the acoustic signal was more favorable in the regime that was under pressure than during air compression. In the following, we adopted the maximum test laser pulse energy (30 mJ), corresponding to the maximum acoustic signal intensity and speed of the laser-induced shockwave, where the largest differences in the acoustic signal between explosives and other materials were expected [37].

**Figure 3:**
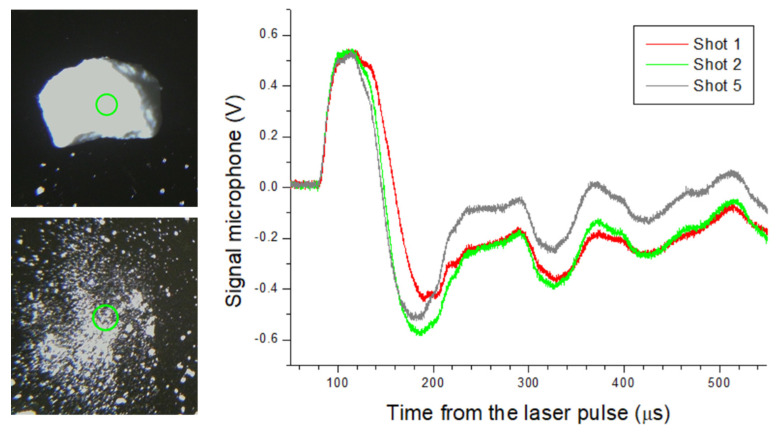
(**Left**): Photos of RDX crystal before and after the first laser pulse (30 mJ) where the laser spot is encircled. (**Right**): Microphone signal for the laser shots 1—on RDX crystal, 2—on pulverized RDX, and 5—on cleaned substrate.

In all measurements on the residues, the substrate was fully cleaned by the first 1–3 laser shots at the same position, leading to a stable acoustic signal for the successive pulses, initially checked up to 10 laser pulses. In the case of a large sample grain inside the laser spot, as in the example of the RDX crystal shown in Figure 3, the sample–laser interaction for the first laser pulse was equivalent to the probing of the bulk sample. Due to a small sample volume, the first laser pulse disintegrated the crystal, and the successive laser shot interacted with residues of fine powder. The acoustic signal on the RDX crystal (first laser shot) showed a slower risetime, enlarged wavefront, and smaller amplitude compared with the pulverized RDX (second laser shot) and the cleaned substrate (fifth laser shot); the slower risetime of the acoustic signal was characteristic for combustion rather than for explosion. This finding indicates that the sample interaction with a laser pulse with relatively low energy, as shown here, is enhanced by the radiation-absorbing silica substrate, making this material favorable for the detection of trace materials.

### 3.2. Discrimination of Explosives in Traces

**Figure 4:**
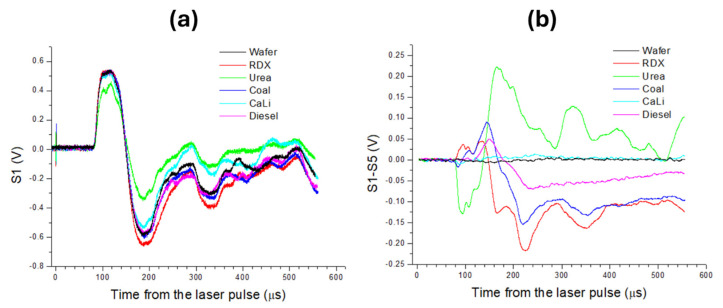
Comparative microphone signals for clean wafer and the same for particles of RDX, urea nitrate, coal, mixed Ca and Li carbonates, and smeared residues of diesel: (**a**) captured after the first laser shot (S1) in sequence of five shots; (**b**) obtained by subtraction of the wafer’s contributions as ΔR = S_1_ − S_5_ and smoothed by over 50 points.

In the following, we considered that the microphone signal from residue S_R_ corresponded to the first or second laser pulse (if different from the substrate’s signal) delivered to the traces on the wafer. Due to the high sampling rate (10 MHz) compared with the dynamic range of the microphone (20 KHz), in the following, we considered the S_R_ signal averaged over 50 consecutive points. Examples of the microphone signal from five classes of material are shown in Figure 4a. Among the reported cases, the largest amplitude corresponded to the RDX residue, where this was more evident by observing the first negative peak, as expected from Figure 2b. The lowest signal amplitude here was obtained for urea nitrate particles; also, for Ca-Li carbonate powder, the signal amplitude was significantly lower than for the clean substrate. Apparently, the signals from coal and diesel were very similar to those from the wafer, but the differences became evident after subtracting the signal from shot 5 (S_5_) taken during the same measurements (Figure 4b). In the following, we adopted the signal difference between the residue and the wafer as ΔR = S_R_ − S_5_. This subtraction, relative to the substrate cleaned by the previous four pulses, eliminated variations due to small changes in the sample/holder positioning from one measurement to another and cut the features caused by echoes. From Figure 4b, it could be observed that, regarding explosives, the initial signal difference ΔR, starting after about 84 µs from the laser pulse, was positive, indicating a faster pressure wave than for the clean wafer.

Laser ablation of high explosives produced more intense exothermic chemical reactions than the low-energetic or non-energetic materials, thus increasing the velocity of laser-induced air shock front [37,41,42]. Here, for non-explosive substances, the shock front was slower than for explosives, as visible from the initial negative value of ΔR. This slowing of the wavefront was particularly pronounced after placing urea nitrate particles on the wafer, where ΔR also assumed large positive after transition of the compression wave. The addition of Ca-Li carbonate particles on the wafer produced very little alteration of the acoustic signal. The width of the first pressure wave, calculated between the time when the signal S_R_ reached 0.1 V and successively dropped to zero, was between 61 µs and 68 µs for non-explosive substances, while for explosives the front width was enlarged up to 75 µs. This widening of the pressure wave caused a forward shift of the first minimum position in S_R_; for non-explosives its location was between 184 and 189 µs, while for explosives the valley was positioned up to 204 µs. However, due to a relatively wide negative peak, calculation of its position produced large errors.

The longer duration of the pressure wave for explosives compared with other sample classes indicated a major release of energy through fast chemical reactions triggered by the laser. The relatively large distance between the plasma and the microphone, necessary to avoid signal saturation, corresponded to the detection of the pressure wave that was already slowed down. The prolonged under-pressure period, observed for explosives, coal, and diesel after the shockwave front, might have been caused by post-shock-sustained combustion. This part of the acoustic signal could be linked to material-specific effects, e.g., density and energy release rates, that in our case also depended on the amount of material involved in plasma formation.

**Figure 5:**
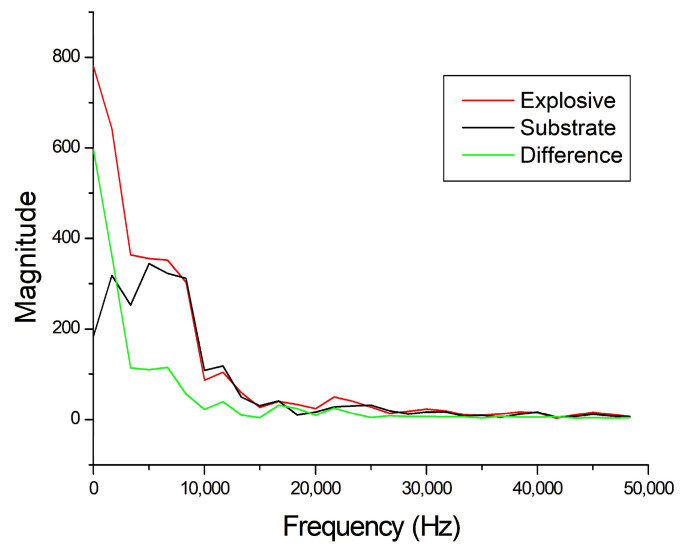
Example of FFT analysis on RDX particles (red line) and clean substrate (black line), where their difference is also shown (green line).

An example of FFT analysis of acoustic signals generated on RDX by laser shot 1 and the cleaned substrate at the same position (laser shot 5), is shown in Figure 5. As it can be seen, there was dominance in the low-frequency region of the signal from the explosive; this is a strong indicator of combustion or detonation processes, as these phenomena release energy over a broad time scale, contributing to lower frequencies. Post-detonation phenomena, such as rarefaction waves, also tend to manifest in this frequency range. The absence of high-frequency features here was also expected due to the distance between the laser spot and the microphone, the cut-off of high frequencies (>20 KHz) by the used acoustic detectors, and the impossibility of denoising the acoustic spectrum through averaging multiple measurements on the trace materials.

**Figure 6:**
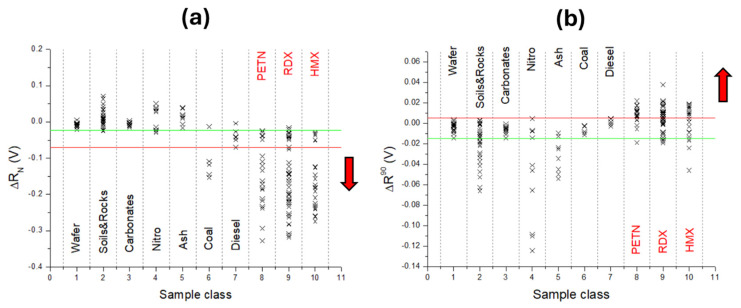
(**a**) The maximum negative signal difference ΔR_N_ with respect to wafer found for different material classes. (**b**) The signal difference with respect to wafer measured at a delay of 90 µs from the laser pulse. Green lines indicate limits for the inert samples; red lines are the limits placed for explosives. The arrow indicates direction with increasing mass or differentiation of explosives.

In the following, the first parameter that we considered for distinguishing explosives from other residues was the largest signal difference ΔR_N_ in under-pressure regions that showed similarity with the first peak amplitude, i.e., shockwave intensity, but without saturation of the detector (Figure 6a). This feature was expected to increase in the presence of rapidly oxidizing substances, e.g., explosives. The value (ΔR_N_) was calculated as the first minimum in ΔR starting from 160 µs after the laser pulse. The inert materials (wafer, soils, rocks, carbonates, ash) had ΔR_N_ > −0.023 V, while larger negative values occurred for nitro compounds, particularly for explosives, then for coal and diesel. If placing the limit for separating explosives from diesel (ΔR_N_ > −0.071 V), below this value we found 135 (out of 171) points acquired for explosives and 6 (of 10) points acquired for coal. These findings indicate that regimes generated under pressure are related to the combustion process, particularly considering that, different to coal, the points acquired for ash do not compromise the identification of explosives. Certain materials (dust, powders, gases, or volatile organic liquids) may be combustible or flammable under ordinary conditions but become explosive in specific situations or forms, such as dispersed airborne clouds, or in the case of confinement or sudden release. For example, the explosive performances of coal particles are studied in pulse detonation engines, with the aim of achieving more efficient and clean coal utilization [43].

The analog results shown in Figure 6a were obtained by calculating ΔR^M1^—the mean value of ΔR in the interval 180–300 µs after the laser pulse where the largest differences among the substances were expected (see Figure 4b). In this way, the calculation error was reduced compared with a single peak value. It is important to note that the points measured for explosives and falling above the red separation line in Figure 6a might be attributed to very low amounts of sample inside the laser spot.

Previously, we found that the first risetime of the signal S_R_ was generally faster for explosives than for other residues, as expected due to the initial rapid exothermic reactions of explosives. To account for this while reducing the calculation errors and slight signal dependency on the relative sample position, we considered the values ΔR^90^ as the measured signal difference at a delay of 90 µs, i.e., soon after the arrival of the pressure wave from the clean wafer (≈84 µs). The results shown in Figure 6b indicate that the addition of inert substances onto the wafer slowed down the acoustic signal. By increasing the surface coverage, the laser–target interaction was reduced [39], the generated plasma was weaker, and the consequent acoustic signal was therefore less intense. The values of ΔR^90^ found for coal were grouped towards the lower limit for clean wafer, while the opposite occurred for the diesel residue. Only for explosives, the values of ΔR^90^ exceeded the upper limit for the wafer, indicating a faster propagation of the laser-induced shockwave. If attributing the detection of explosives to the points above the wafer’s limits of ΔR^90^ > 0.051 V, 93 points (of 171) generated for explosives fell into this zone.

**Figure 7:**
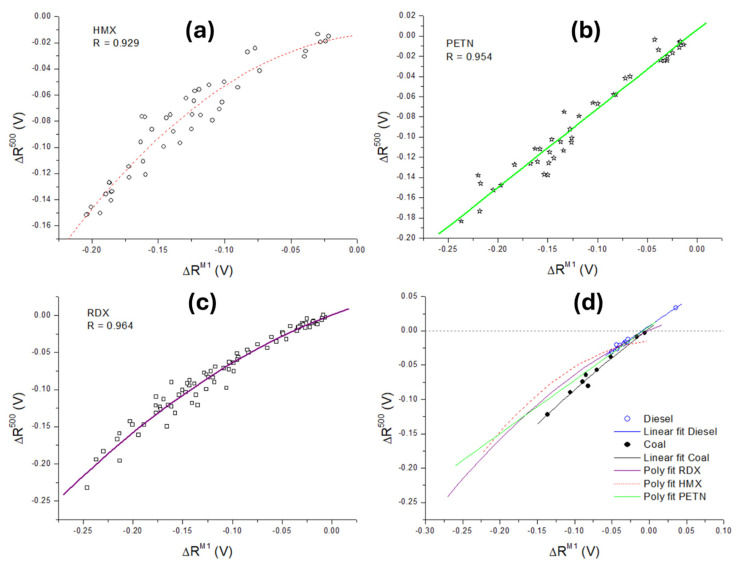
(**a**) Dependency of the average signal difference with respect to wafer, measured in interval 500–550 µs (ΔR^M500^), as a function of the same in interval 180–300 µs (ΔR^M1^), for: (**a**) HMX, polynomial fit 2nd order; (**b**) PETN, linear fit; (**c**) RDX, polynomial fit 2nd order; (**d**) coal (black, 2nd order polynomial fit); and diesel (blue, linear fit) in comparison with the fitted data for explosives.

Another feature was examined to distinguish the samples with regards to the signal recovery after the first minimum, which was expected to reflect sustained combustion in the plasma. Given that the microphone’s minimum here was between 180 and 240 µs from the laser pulse and that the difference between explosives and the wafer was persistent, we compared the signal difference ΔR^M500^ averaged between 500 and 550 µs to ΔR^M1^, previously defined as the mean signal difference in the interval 180–300 µs. For the three tested explosives, this dependency is shown in Figure 7a–c and approximated by linear or second order polynomial fit. The left side of the graphs corresponds to higher amounts of explosives inside the laser spot, where the difference ΔR in the signal from the residue and the wafer is generally higher. By decreasing the sample amount, ΔR tends to zero (right side of the graphs). In Figure 7d, the fit functions ΔR^M500^(ΔR^M1^) for explosives are compared with the data obtained for diesel (linear fit) and coal (polynomial fit). It could be observed that, for non-explosives, the points were grouped towards smaller signal differences, indicating the prevalence of sustained combustion with respect to fast exothermic reaction. Among the explosives, HMX showed the most pronounced curve bending with a reduction in sample amount.

We also observed different behaviors of the positive peak in ΔR occurring between the time when the pressure wave was raised to 90% of the maximum and the successive first minimum, after 180 µs or longer from the laser pulse. The positive peak value in this time interval was signed as ΔR^P^, but its dependency on the other measured values was scattered and not depicted graphically here. This peak reflected the maximum acoustic wave intensity, dependent on the fast energy release in the plasma.

**Figure 8:**
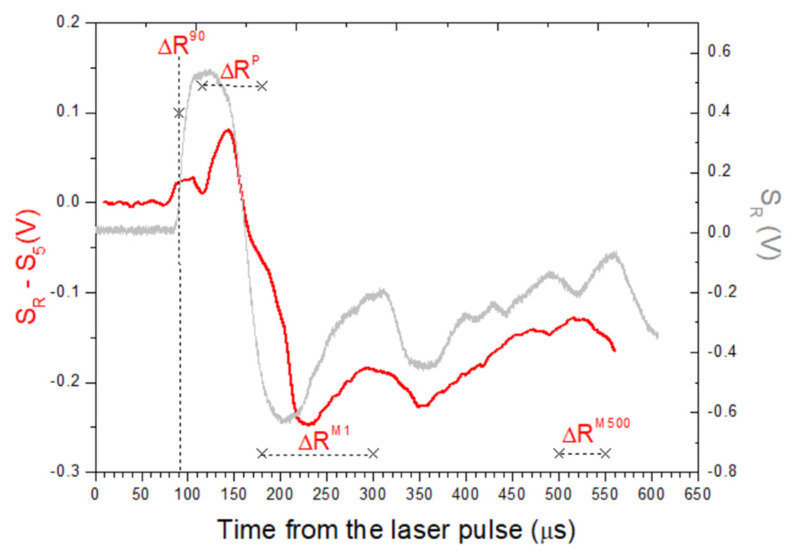
Features in the signal difference between residue and wafer (red line) used for classification of explosives: ΔR^M90^- value at delay od 90 µs, ΔR^P^- peak value between 115 and 180 µs, ΔR^M1^- mean value between 180 and 300 µs, and ΔR^M500^- mean value between 500 and 550 µs. The original microphone signal S_R_ from residue is comparatively shown (gray line).

**Figure 9:**
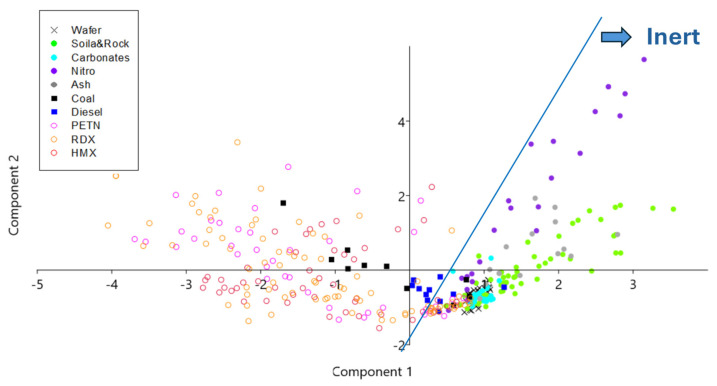
PCA projection for the first two components where the whole sample set (Classes 1–10) was included. The blue line indicates the separation between inert and burning/explosive materials.

Four characteristic signal parameters, namely ΔR^90^, ΔR^P^, ΔR^M1^, and ΔR^M500^, illustrated in Figure 8, were considered in principal component analysis (PCA), performed by PAST software (version 326b). The dataset included all 10 sample classes, and the scores obtained for the first three PCA components were 62.8%, 34.3%, and 2.4%, which together covered 99.5% of variability. The PCA projections provided the complete separation of inert sample materials from explosives, coal, and diesel if they were present in a sufficient amount inside the laser spot (Figure 9).

In the following, we applied a linear discriminant analysis (LDA) classifier, where each data point was assigned to the group that gave a minimal Mahalanobis distance to the sample group (class) mean. In the obtained LDA confusion matrix (Table 2), where off-diagonal counts indicated the number of points incorrectly attributed to another sample class, the false positives, i.e., false classifications, as explosives (Classes 8–10) were fully absent. The points measured for explosives with an insufficient sample mass fell into the lower classes (1–7). About 90% of points acquired for HMX and identified as explosives (Classes 8–10) fell exactly in the correct class (Class 10); differently, for PETN (Class 8) and RDX (Class 9), the correct class attribution was much lower, and the data points were distributed among Classes 8–10. The differentiation of HMX from the other two explosives could be explained by a higher density and detonation velocity (9100 m/s compared with 8400 m/s for PETN and 8600 m/s for RDX). It is interesting to observe that coal (Class 6) and diesel (Class 7) were or correctly classified, or the points fell into Class 1 (wafer) if an insufficient amount of sample was inside the laser spot.

### 3.3. Limits of Detection of Explosives

To place a basis for the determination of mass of particles for explosives inside the laser spot, we exploited the LIBS spectra from nano-plotted samples, acquired simultaneously with the acoustic signal. The LIBS acquisition delay from the laser pulse was 2 µs, while the gate was set to the minimum (30 µs). The measurements were performed on RDX spots with masses of 1, 3, 10, and 30 ng, and on PETN spots with masses 1, 3, 13, and 30 ng. For each sample mass, the signal was acquired from eight plotted spots and the line intensity from C I line at 247.86 nm was considered as the peak integral resulting from fitting by Voigt’s function. Comparative measurements were performed also on the clean wafer substrate, where a very weak emission from C I was also observed.

**Figure 10:**
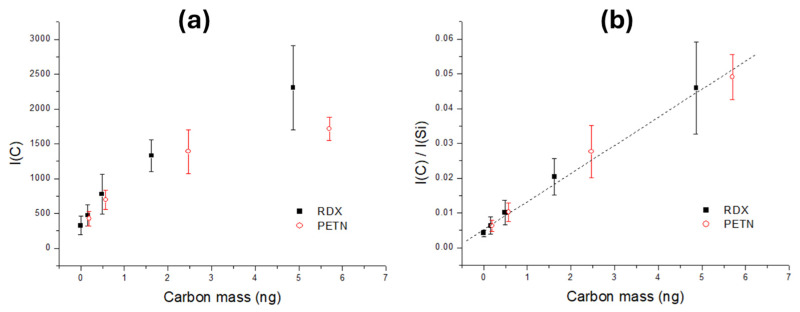
(**a**) Intensity of C I line as a function of carbon mass in printed RDX (black) and PETN (red) samples. (**b**) C I peak intensity normalized on Si I line where the linear fit corresponds to the combined data from both samples.

From the chemical formulas of RDX and PETN, the corresponding carbon represented 16.22% and 18.99% of the total sample mass, respectively. Dependency of the C I peak intensity on the carbon mass in the samples tended to saturation above 2 ng (Figure 10a). The tendency of line peak saturation with an increase in the sample mass on the wafer was already reported [44], also in the absence of self-absorption of the analytical line. This effect is explained by the reduction in the overall plasma intensity and its temperature when increasing the coverage of the Si wafer, the material that enhanced the laser–target coupling. However, by normalizing the C I peak on the Si I peak at 288.16 nm, the dependency on carbon mass could be considered linear in the examined range (Figure 10b), with the Pearsons’ coefficient of 0.995. This linear dependency was further exploited to estimate the masses of the explosive particles from the laser pulse.

In the case of PETN, after removing the points classified as non-explosives by LDA, the minimum peak ratio C/Si in this reduced dataset was 0.028. Based on the linear fit in Figure 10b and the molecular composition, the minimum mass of the detected PETN was 15 ng; the corresponding value ΔR^M1^ was −0.014 V. In a similar way, by eliminating the RDX points classified as not explosive, the minimum C/Si ratio among the remnant points corresponded to a RDX mass of 9.6 ng and to ΔR^M1^ < −0.0095 V. In the case of HMX, the elimination of points not classified as explosives led to ΔR^M1^ values below −0.067. Knowing that the carbon mass in HMX molecules was 16.2%, from the C/Si values, the minimum detected mass was 18 ng.

**Figure 11:**
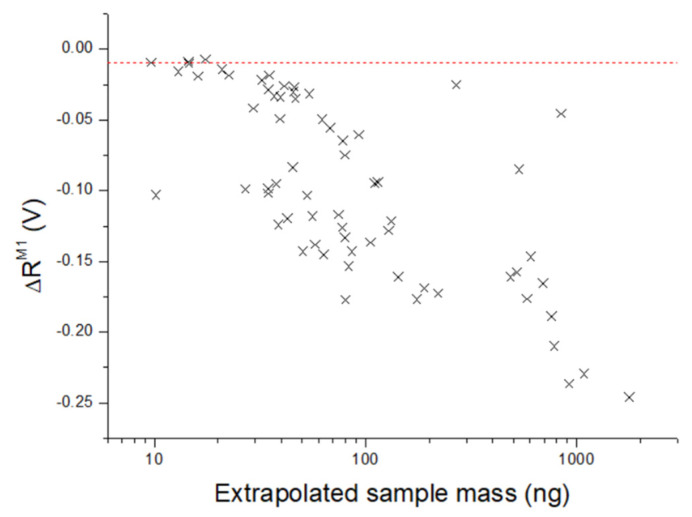
The mean signal difference ΔR^M1^ with respect to clean substrate in the interval 180–300 µs, as a function of the extrapolated RDX mass inside the laser spot. The red line indicates the limit below which all points are classified as explosive.

Due to signal enhancement by the wafer, the classification of explosives depended also on the sample distribution inside the laser spot, making it difficult to establish net detection limits. In Figure 11 we show the measured value of ΔR^M1^ as a function of the extrapolated RDX mass, where the large data scattering indicates both the effect of the signal enhancement by the substrate and missing immediate evaporation of all particles initially present in the laser spot. Such expelled particles could be successively heated by decaying plasma, producing slower and delayed chemical reactions that are reflected in the shape and intensity of the acoustic signal. In the reported case, all points of RDX with the extrapolated mass above 18 ng were classified as explosives, while in the range 9.6–18 ng, the correct classification depended on particle distribution on the target. Note that the mass calibration here was limited to about 30 ng of RDX; thus, above this value, the extrapolated mass was only indicative. The sample points corresponding to the full substrate coverage, without Si emission in the LIBS spectra, were excluded from Figure 11 for obvious reasons.

**Figure 12:**
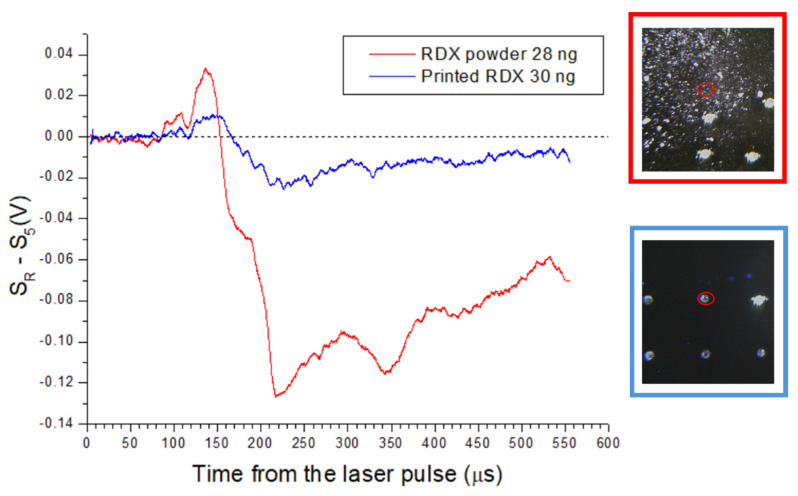
Signal difference with respect to wafer for plotted RDX 30 ng (blue) and RDX powder (red) with estimated mass of 28 ng inside the laser spot. The corresponding photos before the laser shot are also shown where the laser spot is encircled.

**Figure 13:**
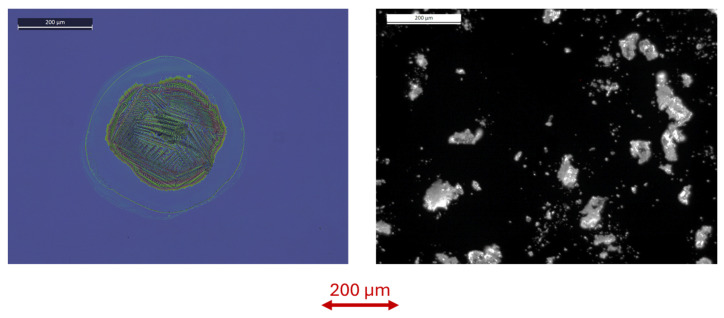
Photo under microscope of the printed RDX spot with mass of 30 ng (**left**) and of RDX powder (**right**).

On the printed samples containing RDX or PETN, the classification through microphone signal was not successful. This was true also on the points containing 30 ng of plotted RDX, the mass that was well above the detection threshold for RDX particles, found to be 9.6 ng or less. The comparative signal difference ΔR for RDX plotted as 30 ng spot and RDX in powder with similar estimated mass, is shown in Figure 12. The observed differences in laser–sample interactions for the powdered and the wet-transferred material could be explained by the modified shape and distribution of the particles, as visible under microscope (Figure 13). By assuming the material density of 1.78 g/mL, the powdered RDX particle of 28 ng could be approximated as a sphere with a diameter of ≈31 µm. On the other hand, the printed RDX spot with 30 ng mass had a diameter of ≈200 µm plus a coffee ring with a low sample content. This printed, very flat RDX spot with height <1 µm, had a large contact area with the substrate, making it more difficult to detach and vaporize the residue by the laser pulse compared with sparse three-dimensional RDX particles/crystals.

Here, estimated detection limits for explosives were obtained in laboratory conditions, in the presence of common reports (power supplies, air-conditioning, fume aspirator, speaking among the personnel, etc.). Due to a very intense acoustic signal generated by the laser-induced plasma and short distance between the plasma and the unidirectional microphone, we did not observe any effect from the surrounding noise on the measurements. However, in view of making a portable instrument for the outdoor detection of explosives in traces, the occurrence of intense uncontrolled external acoustic signals should be considered. The technical solutions for transferring the instrument from a common to an uncontrolled operating environment might include acoustic isolation of the laser–target interaction and detection cell, or the use of multiple microphones for extrapolating the surrounding noise, to be subtracted from the laser-induced signal.

## 4. Conclusions

In this work we demonstrated that the acoustic signal from particles placed on silica wafers differed between inert samples, explosives, and materials with burning potential. For explosives, the acoustic signal had a faster rise, larger amplitude, and different width and attenuation compared with the other sample classes. These differences were particularly evident when analyzing the signal from residue after subtraction of the same after four cleaning laser pulses to eliminate the echoes from nearby components. We found four different features in the subtracted signal that allowed recognition of explosives without the presence of false positives. Furthermore, HMX characterized by high detonation velocity with respect to PETN and RDX, was recognized in 90% of cases, while coal and diesel were also correctly classified when present in sufficient amounts inside the laser spot.

The silica wafer substrate enhanced the plasma–sample interaction, dependent on residue distribution inside the laser spot. Consequently, the detection threshold for residues was not neat. However, the lowest detected mass of PETN, RDX, and HMX by the acoustic signal was 15 ng, 9.6 ng, and 18 ng, respectively. The explosives printed on wafers through the wet transfer showed very flat distribution on the substrate, where the large contact area prevented the efficient laser vaporization and detection for the tested masses up to 30 ng.

Here, obtained detection sensitivity through the laser-induced acoustic signal on trace explosives is in line with other portable instruments, e.g., atmospheric MS, IMS, and SERS. The advantage of the proposed approach regards the absence of false positives, and cheap detectors with a frequency signal (<20 KHz) might be captured by a low-resolution digital oscilloscope, low cost, and a stable substrate compared with SERS, and a possibility of integration with LIBS spectrometers for elemental characterization of samples. The selectivity of the method should be further studied, where the obtained results suggest that the identification of explosives in traces will not be univocal, as in the case of MS or SERS for pure substances, but dependent on the chemical energy of the material.

Future developments include testing a larger number of substances with chemical energy than here to verify the capability of the method to distinguish, besides explosives, other various flammable substances. The sensor development should regard the scaling of the laser pulse energy, with a simultaneous reduction in the distance between the plasma and the microphone, to capture the acoustic signal without saturation and before the laser-induced shockwave is importantly slowed down by the surrounding air.

## Figures and Tables

**Table 1:** Sample set for testing and for mass calibration.

Test Set			
Class Number	Class Type	Specification	Form
1	Wafer	Clean	Solid
2	Soils and rocks	NIST2709 NIST2710 NIST2711 NCS DC73302	Powder
3	Carbonates	50:50 CaCO_3_:BaCO_3_ 90:10 CaCO_3_:LiCO_3_	Powder
4	Nitrates	KNO3 Urea nitrate	Powder/crystals
5	Coal fly ash	NIST 1633a	Powder
6	Bituminous coal	NIST 1632b	Powder
7	Diesel	For car vehicle	Smeared film
8	PETN	Origin 1 Origin 2	Powder/crystals
9	RDX	Origin 1 Origin 2 Origin 3	Powder/crystals
10	HMX	Origin 1 Origin 2	Powder/crystals
**Calibration set**			
**Class**	**Class Type**	**Specification**	**Form**
P1	PETN	1, 3, 13 and 30 ng per spot	Nano-plotted dots
P2	RDX	1, 3, 10 and 30 ng per spot	Nano-plotted dots

**Table 2:** Confusion matrix for LDA classification algorithm applied to wafer and nine sample classes placed on wafer where explosives are in red.

Class	1	2	3	4	5	6	7	8	9	10	Total
**1**	**21**	0	11	0	0	0	2	0	0	0	34
**2**	9	**19**	16	7	6	0	1	0	0	0	58
**3**	9	1	**13**	0	1	0	1	0	0	0	25
**4**	3	0	2	**12**	4	0	2	0	0	0	23
**5**	2	3	1	4	**4**	0	2	0	0	0	16
**6**	2	0	0	0	0	**8**	0	0	0	0	10
**7**	2	0	0	0	0	0	**9**	0	0	0	11
**8**	2	0	2	0	0	1	8	**17**	9	2	41
**9**	7	0	4	0	1	3	14	22	**16**	16	83
**10**	1	0	2	0	0	0	5	0	4	**35**	47
**Total**	58	23	51	23	16	12	44	39	29	53	**348**

## Data Availability

The data of the measurements that support the findings of this study are available from the corresponding author upon reasonable request.

## References

[1] Zapata F., Garcia-Ruiz C. (2021). Chemical classification of explosives. Crit. Rev. Anal. Chem..

[2] Ewing R.G., Waltman M.J., Atkinson D.A., Grate J.W., Hotchkiss P.J. (2013). The vapor pressures of explosives. TrAC Trends Anal. Chem..

[3] Yinon J., Marshall M., Oxley J.C. (2009). Analysis and detection of explosives by mass spectrometry. Aspects of Explosives Detection.

[4] Santos J., Galceran M.T. (2003). Modern developments in gas chromatography–mass spectrometry-based environmental analysis. J. Chromatogr. A.

[5] Mary Celin S., Sharma B., Bhanot P., Kalsi A., Sahai S., Tanwar R.K. (2022). Trends in environmental monitoring of high explosives present in soil/sediment/groundwater using LC–MS/MS. Mass Spectrom. Rev..

[6] Gross J.H. (2014). Direct analysis in real time—A critical review on DART-MS. Anal. Bioanal. Chem..

[7] Frazier J., Benefield V., Zhang M. (2020). Practical investigation of direct analysis in real time mass spectrometry for fast screening of explosives. Forensic Chem..

[8] Ewing R.G., Atkinson D.A., Eiceman G.A., Ewing G.J. (2001). A critical review of ion mobility spectrometry for the detection of explosives and explosive-related compounds. Talanta.

[9] DeBono R., Lareau R.T., Kagan A., Oxley J.C. (2022). Trace detection of explosives by ion mobility spectrometry. Counterterrorist Detection Techniques of Explosives.

[10] López-López M., Garcia-Ruiz C. (2014). Infrared and Raman spectroscopy techniques applied to identification of explosives. TrAC Trends Anal. Chem..

[11] Abdallah A., Mahmoud A., Mokhtar M., Mousa A., Ayoub H.S., Elbashar Y.H. (2022). Raman spectroscopic and advanced signal processing analyses for real-time standoff detection and identification of explosives. Opt. Quantum Electron..

[12] Lazic V., Palucci A., De Dominicis L., Nuvoli M., Pistilli M., Menicucci I., Colao F., Almaviva S. (2019). Integrated laser sensor (ILS) for remote surface analysis: Application for detecting explosives in fingerprints. Sensors.

[13] Logrado L.P.L., Ferreira da Silva B.M., da Silveira Neto B.A. (2025). Assessment of handheld Raman spectroscopy for forensic analysis of dark-colored bulk explosive fuel–oxidizer mixtures. J. Forensic Sci..

[14] Gillibert R., Huang J.Q., Zhang Y., Fu W.L., Lamy de la Chapelle M. (2018). Explosive detection by surface-enhanced Raman scattering. TrAC Trends Anal. Chem..

[15] Muehlethaler C., Leona M., Lombardi J.R. (2016). Review of surface-enhanced Raman scattering applications in forensic science. Anal. Chem..

[16] Bharati M.S.S., Soma V.R. (2021). Flexible SERS substrates for hazardous materials detection: Recent advances. Opto-Electron. Adv..

[17] Wasilewski T., Gębicki J. (2021). Emerging strategies for enhancing detection of explosives by artificial olfaction. Microchem. J..

[18] Moore D.S. (2004). Instrumentation for trace detection of high explosives. Rev. Sci. Instrum..

[19] Caygill J.S., Davis F., Higson S.P.J. (2012). Current trends in explosive detection techniques. Talanta.

[20] Chuen To K., Ben-Jaber S., Parkin I.P. (2020). Recent developments in the field of explosive trace detection. ACS Nano.

[21] Klapec D.J., Czarnopys G., Pannuto J. (2020). Interpol review of detection and characterization of explosives and explosives residues 2016–2019. Forensic Sci. Int. Synergy.

[22] Al-Fakih A.M., Nabat K.Y., Jiang T., Liu L. (2025). Recent innovations in explosive trace detection: Advances and emerging technologies. Trends Environ. Anal. Chem..

[23] Augustyniak D., Szala M. (2025). Field explosives detectors—Current status and development prospects. Sensors.

[24] Lazic V., Andreoli F., Almaviva S., Pistilli M., Menicucci I., Ulrich C., Schnürer F., Chirico R. (2024). A novel LIBS sensor for sample examinations on a crime scene. Sensors.

[25] De Lucia F.C., Harmon R.S., McNesby K.L., Winkel R.J., Miziolek A.W. (2003). Laser-induced breakdown spectroscopy analysis of energetic materials. Appl. Opt..

[26] Gottfried J.L., De Lucia F.C., Munson C.A., Miziolek A.W. (2009). Laser-induced breakdown spectroscopy for detection of explosives residues. Anal. Bioanal. Chem..

[27] Lazic V., Palucci A., Jovicevic S., Poggi C., Buono E. (2009). Analysis of explosive and other organic residues by laser-induced breakdown spectroscopy. Spectrochim. Acta B.

[28] Lazic V., Palucci A., Jovicevic S., Carpanese M. (2011). Detection of explosives in traces by laser-induced breakdown spectroscopy. Spectrochim. Acta B.

[29] Abdelhamid M., Fortes F.J., Harith M.A., Laserna J.J. (2011). Analysis of explosive residues in human fingerprints using optical catapulting–laser-induced breakdown spectroscopy. J. Anal. At. Spectrom..

[30] Moros J., Serrano J., Gallego F.J., Macías J., Laserna J.J. (2013). Recognition of explosives fingerprints using machine learning and LIBS. Talanta.

[31] Ding J., Zhang T., Li H. (2023). Recent advances in LIBS for explosive analysis. TrAC Trends Anal. Chem..

[32] Alvarez-Llamas C., Purohit P., Moros J., Laserna J. (2022). LIBS-acoustic fusion for mineral differentiation. Anal. Chem..

[33] Bosáková M., Novotný K., Moros J., Laserna J. (2025). Acoustic signal in overcoming the matrix effect in LIBS. Spectrochim. Acta B.

[34] Lu P., Zhuo Z., Zhang W., Tang J., Xing T., Wang Y., Sun T., Lu J. (2021). Determination of calorific value in coal by LIBS with acoustic normalization. Appl. Phys. B.

[35] Hargather M.J., Winter K.O., Kimberley J., Wei T. (2023). Shock wave scaling. Shock. Waves.

[36] Gottfried J.L. (2014). New Laboratory-Scale Method for the Determination of Explosive Performance from Laser-Induced Shock Waves.

[37] Gottfried J.L., Wainwright E.R. (2023). Laser-induced air shock from energetic materials (LASEM). J. Energ. Mater..

[38] Wynn C.M., Haupt R.W., Doherty J.H., Kunz R.R., Bai W., Diebold G. (2016). Use of photoacoustic excitation and laser vibrometry for remote explosives detection. Appl. Opt..

[39] Lazic V., De Ninno A. (2017). Calibration approach for extremely variable laser induced plasmas and a strategy to reduce the matrix effect in general. Spectrochim. Acta B.

[40] Vinić M., Aruffo E., Andreoli F., Ivković M., Lazic V. (2020). Quantification of heavy metals in oils with μL volume by laser induced breakdown spectroscopy and minimazing of the matrix effect. Spectrochim. Acta Part B.

[41] Wainwright E.R., Dean S.W. (2025). Acoustic measurements of laser-induced microshocks: Time of arrival to yield estimations. Shock Waves.

[42] Wang X., Liu R., He Y., Fu Y., Wang J., Li A., Guo X., Wang M., Guo W., Zhang T. (2022). Determination of detonation characteristics by laser-induced plasma spectra and micro-explosion dynamics. Opt Express..

[43] Guo J., Shirong G. (2025). Preliminary study on the explosive performance of coal dust and prospects for engineering applications. Sci. Rep..

[44] Lazic V., Markovic M., Stankov B.D., Andreoli F., Ulrich C., Kuzmanovic M. (2026). Calibration Coefficients for semi-quantitative analysis by LIBS in samples containing Calcium. Spectrochim. Acta Part B.

